# Hospital and Health News

**Published:** 1924-06

**Authors:** 


### HOSPITAL AND HEALTH NEWS.

### In Puris Naturalibus.

"I am always naked when I shave."—*Sir Herbert Barker.*

### Royal Patrons of the London Homœopathic Hospital.

The Prince of Wales has become patron, and the Duke of York president, of the London Homœopathic Hospital.

### Queen Charlotte's Hospital.

A Supplemental Royal Charter has been granted changing the name of Queen Charlotte's Lying-in Hospital to Queen Charlotte's Maternity Hospital. The Supplemental Charter also provides for the election of ladies equally with men on the Committee of Management.

### How They Die in New Elvet.

In the New Elvet area of Durham the infant death rate is 247 per 1,000 births, which is over 100 per cent. higher than the rate for Durham City generally. With regard to the general death rate in the New Elvet area more than one out of every five persons dies.

### "Bisto" Sickfeeder.

With reference to a paragraph in our last issue about the "Bisto" Sickfeeder, manufactured by Messrs. Bishop and Stonier, we would point out that the figures, 30S., 36S. and 42S., refer, of course, to the sizes and not to the prices of the feeders. Pottery articles are still called by the old-fashioned trade method of numbers where sizes are made.

### The Relay and the Hospital.

A little pamphlet which will probably be of interest to hospital authorities is published by the Relay Automatic Telephone Company, Ltd., entitled "The Relay and the Hospital." The Company will be glad to send a copy of the pamphlet to any of our readers who apply to the Company at Marconi House, W.C. 2.

### Edinburgh Royal Infirmary.

The proposed extension of the Edinburgh Royal Maternity and Simpson Memorial Hospital may take the form of a new institution. A committee is now considering a suitable site, and the Scottish Board of Health supports the proposal. Dr. Nasmyth, chairman of the directors, has stated that an up-to-date hospital with 100 beds, costing £100,000, is needed, and that a large scheme will be launched shortly.

### A Help to the Deaf.

Deaf people may find their hearing considerably assisted by the use of the "Ardente Acoustique," which is said to remove strain, conveying sounds in a natural manner, and thus improving and conserving the hearing. This Acoustique is approved by many leading aurists, and is inconspicuous in use. It is the invention of Mr. R. H. Dent, and is manufactured at 95 Wigmore Street, W. 1. It is also exhibited at Stand No. 994 in the Palace of Industries at Wembley.

### Two Resignations.

Mr. H. H. Bedford has resigned the chairmanship of the Sheffield Royal Infirmary, which he thinks should be in the hands of a younger man. This follows Mr. Walde's retirement from the chair of the Royal Hospital, and thus removes two noted hospital workers from the active list. Mr. Bedford succeeded Mr. J. Marshall in 1913, at which time he had been for almost six years a member of the board, as his father had been before him.

### The Ranyard Mission.

The annual meeting of the Ranyard Mission will be held on Friday, May 30, at 3 p.m., in the Central Hall (small), Westminster, when the chairman will be the Bishop of Barking. The Sixty-seventh Birthday Celebration for the workers will be held immediately after the meeting. In connection with this Celebration there will be a Pound Day, and gifts in money or kind (preferably tea and sugar) will be gratefully received at the Hall on May 30, or at Ranyard House, 25 Russell Square, W.C. 1.

### Help for Holiday-makers.

The search for seaside apartments is peculiarly discouraging, and many holiday-makers will be glad of "Cutts's Holiday Apartments Guide," which gives a list of clean, comfortable rooms at moderate prices as well as a number of boarding-houses. The apartments have been inspected by lady representatives, and no pains have been spared to make the guide authentic and up to date. It is on sale at Smith's bookstalls, or can be obtained direct from the publishers, Messrs. Larby, 30 Paternoster Row, E.C. 4.

### New Chairman of Sheffield Royal Hospital.

Mr. F. M. Osborn has succeeded Mr. Philip K. Wake in the chairmanship of the Sheffield Royal Hospital. Since 1912 Mr. Osborn has been a member of the board and has represented the Royal Hospital on the Joint Hospitals' Council, of which he is the vice-chairman. His public work also includes the chairmanship of the Sheffield Interviewing Board of the Appointments Department of the Ministry of Labour. In accepting his new post, Mr. Osborn suggested that a deputy-chairman should also be appointed by the Royal Hospital.

### Modern Hospital Equipment.

Messrs. Manlove, Alliott & Co., Ltd., of Nottingham, the well-known firm of engineers, specialise in mechanical equipment for hospitals, including steam disinfectors and sterilizers, cooking apparatus, incinerators and laundry machinery. Their booklet on The Mechanical Equipment of a Modern Hospital gives illustrations and information as to these manufactures, and Messrs. Manlove, Alliott & Co. will be pleased to send a copy of it to any reader of this journal on application.

### Enlargement of Essex County Hospital.

On May 14th the foundation stones were laid of the new out-patients' block of the Essex County Hospital, Colchester. The Chairman of the Committee, Colonel J. C. Tabor, said that the hospital was started just over a hundred years ago, and that successive committees had made extensions and improvements, but it was still not up to modern requirements and efficiency. So, from amongst many much needed improvements, they had chosen to build a new out-patients' department, where they would also have rooms for paying patients and three for resident officers. The next aim would be the enlargement of the nurses' home.

### Insulin Further Reduced.

Messrs. Burroughs, Wellcome and Co. have made a further substantial reduction in the price of "Wellcome" brand insulin. The new price is 4s. 8d. per phial of 100 units (10 average doses), and there is a special price to hospitals. "Wellcome" brand insulin, as now issued, is of a remarkable degree of purity. The physiological activity of the material, insulin hydrochloride, of which "Wellcome" brand insulin is a standardised solution is such that approximately one milligram (1.64 grain) represents an average human dose of 10 units.

### International Health Work.

One of the results of the recent interchange of public health officers who visited this country under the League of Nations auspices to study English public health methods is the decision to set up an international society open to all medical officers of health who are taking part in the various interchanges organised by the League. A Provisional Committee, composed of doctors from Great Britain, Russia, France, Germany, Poland, Italy and Ecuador, has been elected to draw up the Constitution. The Society will have its headquarters at Geneva, and all public health officers who have already taken part in League Interchanges—240 from forty-three different countries—will be invited to become original members.

### "For a Strenuous Life."

If there are any who, six years after the Great War, still suffer pain and discomfort from their artificial limbs, it must be because they have not had the good fortune to try "Grossmith" limbs. Established 160 years ago, Messrs. W. R. Grossmith, Ltd., of 12 Burleigh Street, Strand, have utilised their long experience in making limbs which, as any-one who cares to write for their catalogue will see, are remarkably light and durable, comfortable for every occupation, whether it be walking, riding or any form of sport; they have been approved by the Ministry of Pensions. They also make "Light limbs for Ladies," a claim which, were it not such a boon, would sound like speaking of a serious matter flippantly.

### Sir Malcolm Morris Memorial Fund.

To perpetuate the memory of Sir Malcolm Morris, K.C.V.O., F.R.C.S., it was resolved at a recent meeting of representatives of the various hospitals and organisations with which he was particularly associated, to endeavour to raise a sum of money sufficient to provide for an annual lecture on the preventive aspects of public health and dermatology. If funds permit it is hoped also to endow a bed for cases of skin disease at St. Mary's Hospital. These objects are known to accord with Sir Malcolm Morris's expressed view that the only fitting memorial to a life of successful endeavour is one that carries on the causes to which that life was devoted. A representative Committee has been formed and contributions should be sent to the Honorary Treasurers, "Malcolm Morris Memorial Fund," at 12 Stratford Place, W. 1.

### International Industrial Hygiene.

The Swiss Committee of Health Experts for the First International Meeting for the study of problems of Industrial Hygiene intimates the names of those who have consented to furnish reports. They are: Industrial Lighting and Eye-strain, M. Gaster, Technical Report; M. Oblath, General Physiopathology; and Dr. Stassen, Lighting in Mines and Miners Eyestrain. Vitiated Atmosphere in Workshops: Prof. L. Hill, Ventilation; M. Kohn-Abrest, Dust and Smoke; and Prof. Lehmann, Gases. Value of Fatigue Tests: M. F. Lee, Chemical Methods, General Criticism of Fatigue Tests; Prof. M. Patrizi, Mechanical and Graphical Methods; and M. Wyatt, Psychological Methods. Requests for further information should be addressed to Prof. Cristiani, Institute of Hygiene, University of Geneva, Switzerland.

### The Late Mr. Charles Larking.

The recent death of Mr. Charles Larking has removed a well-known hospital worker who did great service for Norfolk and Norwich Hospital for nearly a quarter of a century. A vice-president of the hospital at the time of his death, he had been chairman of the board in 1914, and perhaps was the most active spirit in financing the institution. In 1900 he acted as secretary during the absence of Mr. Poole Gabbett at the South African War, and became a member of the board in 1901. During the late war Mr. Larking started the fund for the monthly maintenance of military beds, and this yielded £6,000 per annum for four and a-half years. He was a magnet for attracting gifts to the hospital's funds, and himself a generous donor. He will be badly missed just now as it is suggested that Norwich—which has no general war memorial—should provide the extensions, costing £35,000, that the hospital needs in memory of the citizens who lost their lives.

### King's College Hospital and its Critics.

With reference to the criticisms of Mr. Ingleby Oddie regarding two accident cases which could not be received in the wards of King's College Hospital and were sent on to Lambeth Hospital, the Committee have issued a reply, stating that they "profoundly regret that the closing of the wards through lack of funds to maintain the beds should cause suffering to anyone needing treatment, and deprecate the suggestion that street casualties are sent away because they are not wanted. . . . On both occasions in question the men's surgical wards were over-full, extra beds having been already put up and occupied. The casualty beds were also full. . . . A reference to a return presented to the Ambulance Cases Disposal Committee of King Edward's Hospital Fund will show that, compared with the number of beds available, King's College Hospital receives an abnormally large number of cases from the L.C.C. Ambulance Service, being, in fact, second on the list of all the hospitals in London." The statement goes on to say that, in the cases of the two men in question, both of them received treatment before being sent on to Lambeth, and that they did not die until in one case four and in the other ten days after the accidents.

